# A structural basis for prion strain diversity

**DOI:** 10.1038/s41589-022-01229-7

**Published:** 2023-01-16

**Authors:** Szymon W. Manka, Adam Wenborn, Jemma Betts, Susan Joiner, Helen R. Saibil, John Collinge, Jonathan D. F. Wadsworth

**Affiliations:** 1 MRC Prion Unit at UCL, Institute of Prion Diseases, University College London, London, UK; 2 Department of Biological Sciences, Institute of Structural and Molecular Biology, Birkbeck College, University of London, London, UK

**Keywords:** Neurodegenerative diseases, Structure determination, Protein aggregation

## Abstract

Recent cryogenic electron microscopy (cryo-EM) studies of infectious, ex vivo, prion fibrils from hamster 263K and mouse RML prion strains revealed a similar, parallel in-register intermolecular β-sheet (PIRIBS) amyloid architecture. Rungs of the fibrils are composed of individual prion protein (PrP) monomers that fold to create distinct N-terminal and C-terminal lobes. However, disparity in the hamster/mouse PrP sequence precludes understanding of how divergent prion strains emerge from an identical PrP substrate. In this study, we determined the near-atomic resolution cryo-EM structure of infectious, ex vivo mouse prion fibrils from the ME7 prion strain and compared this with the RML fibril structure. This structural comparison of two biologically distinct mouse-adapted prion strains suggests defined folding subdomains of PrP rungs and the way in which they are interrelated, providing a structural definition of intra-species prion strain-specific conformations.

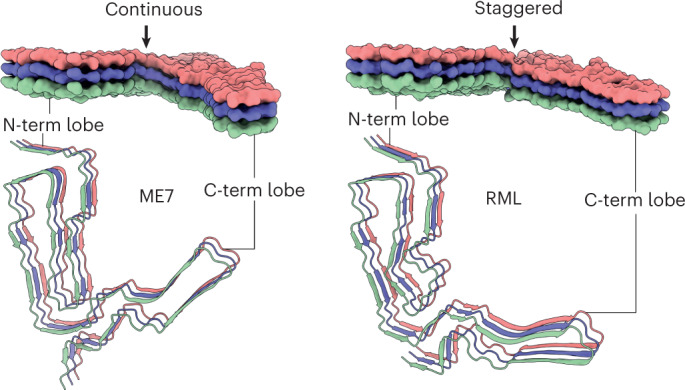

## Abstract (web summary)

A near-atomic resolution strain-specific cryo-EM structure of infectious prion fibrils from mice was determined, revealing a structural definition for intra-species prion strain-specific conformations.

## Main

Transmissible spongiform encephalopathies (TSEs) or prion diseases are invariably fatal neurodegenerative disorders affecting mammals and include Creutzfeldt–Jakob disease (CJD) in humans, scrapie in sheep and goats, bovine spongiform encephalopathy (BSE) in cattle and chronic wasting disease (CWD) in cervids^1–3^. Prions, the causative agent of TSEs, are composed of polymeric fibrillar assemblies of misfolded host-encoded cellular prion protein (PrP^C^) that propagate by fiber elongation and fission. Biologically distinct prion strains can, however, be serially propagated in identical hosts expressing the same PrP^C^ and produce different disease phenotypes. Understanding the structural basis of prion strain diversity is, therefore, of considerable biological interest and evolutionary significance. In addition, prions may transmit disease between mammalian species, as with the infection of humans with BSE prions causing variant CJD. Such cross-species transmission is limited by so-called species barrier effects that relate to structural compatibility of prion strains with host PrP^C^ according to the conformational selection model^2,4^. As it is well recognized that novel prion strains with altered host ranges can arise as a result of PrP polymorphisms in both inter-species and intra-species transmissions^2–6^, determining the structural basis of prion diversity is critical to understanding whether emerging animal prion strains constitute a zoonotic risk to public health^2–7^.

The prion concept has, however, extended well beyond propagation of assemblies of PrP with the discovery of yeast prions^8^. A number of proteins in yeast and other fungi can form prions and demonstrate strain diversity related to well-characterized structural differences^9^; prions of fungi are also not always deleterious to a host^9^. Additionally, all the common neurodegenerative diseases are associated with accumulation of assemblies of host-encoded peptides or proteins, and inoculation studies in suitable transgenic mice have demonstrated the ability of such pathology to be seeded and anatomically spread in a new host, suggesting the involvement of prion-like mechanisms in their pathogenesis^3,10–13^. The relevance of such experimental transmission of the amyloid-β pathology seen in Alzheimer’s disease (AD) to human disease is now clear with the recognition and experimental confirmation of iatrogenic seeding of parenchymal and vascular amyloid-β pathology between humans via cadaver-derived human pituitary extracts^3,14,15^. Indeed, it is now clear that cerebral amyloid angiopathy (CAA) is a prion disease^15^. Assuming, after even longer incubation periods, that such recipients of amyloid-β and tau-contaminated cadaver-derived material also develop a tauopathy in addition to the widespread amyloid-β pathology, thereby meeting the full neuropathological criteria for AD, this would indicate that AD can also result from such iatrogenic exposure to proteopathic seeds and could be considered a prion disease^3,15^. Conformational selection and the general model of prion strains^2,4^ strongly suggests that the prion strain phenomenon will also contribute to the marked phenotypic diversity seen in the commoner neurodegenerative diseases. Indeed, evidence for structural variation in amyloid-β fibrils from distinct clinical subtypes of AD has been reported^12^, and remarkable recent progress with structural characterization of diverse self‐propagating assemblies of tau, amyloid‐β, α‐synuclein and TDP-43 from human brain is now facilitating the detailed exploration of the role for strains in determining phenotype^16–19^.

Although it is now firmly established that mammalian prions causing TSEs are composed of fibrillar assemblies of misfolded host-encoded PrP (classically designated as PrP^Sc^ (ref. 1)) and propagate by means of seeded protein polymerization and fission^1–3,20–24^, the structural mechanisms underpinning prion strain diversity remain unclear. Although it is known that prion strains represent distinct misfolded PrP conformations and assembly states^1–3,25–29^, high-resolution structural definition of prion strains has been extremely problematic. In this regard, because in vitro synthetically generated PrP amyloids are either devoid of detectable prion infectivity or have specific infectivities too low for meaningful structural analysis^2,3,21,30^, efforts to define authentic infectious prion structures have to overcome the difficulty of isolating relatively homogeneous ex vivo prion strain assemblies of correspondingly extremely high specific infectivity suitable for structural analysis^21,31^. Recently, however, significant progress has been made^22–24^.

High-resolution cryogenic electron microscopy (cryo-EM) studies of infectious, ex vivo prions isolated from the hamster 263K prion strain^22^ or the mouse RML prion strain^23^ reported single protofilament helical amyloid fibrils that have a broadly similar, parallel in-register intermolecular β-sheet (PIRIBS) amyloid architecture. Rungs of the fibrils are composed of single PrP monomers that fold to create distinct N-terminal and C-terminal lobes with the N-linked glycans and glycosylphosphatidylinositol (GPI) anchor projecting from the C-terminal lobe^22,23^. Transgenic mice expressing GPI anchorless PrP when infected with RML prions generated in wild-type mice propagate aRML prion fibrils that have the same fold as seen in RML prion-infected wild-type mice^23,24^. The overall architectures of hamster 263K and mouse RML fibrils are remarkably similar and compatible with the defining physicochemical properties of prions^22–24^.

Despite the overall similarity of the hamster 263K and mouse RML prion fibril architectures, there are pronounced differences in the fold of the C-terminal lobes^23,24^, which may be attributable to differences in PrP amino acid sequence and/or distinct conformations associated with divergent prion strains. To determine directly which conformational differences can be attributed to prion strain, comparison of structures of different strains propagated in the same host with identical PrP^C^ substrate is necessary.

Here we report a 2.6-Å cryo-EM structure of fibrils present in highly infectious prion rod preparations isolated from the brain of mice infected with the ME7 mouse-adapted scrapie prion strain^31,32^. Like RML prions^23^, ME7 prion rods are predominantly single protofilament helical amyloid fibrils that coexist with paired protofilaments. Crucially, the fibrils of both mouse prion strains share the same underlying modular architecture but with markedly altered topology. We identified conformationally conserved and variable regions in the N-terminal lobe and a structurally congruent, but differently oriented, disulphide-stapled (DS) hairpin in the C-terminal lobe. ME7 and RML strain diversity appears to be linked to the divergent fold of the conformationally variable region and the orientations of the N-terminal and C-terminal lobes, resulting in distinct helical assemblies.

## Results

### ME7 fibril morphologies resemble those of RML

**Extended Data Fig. 1:**
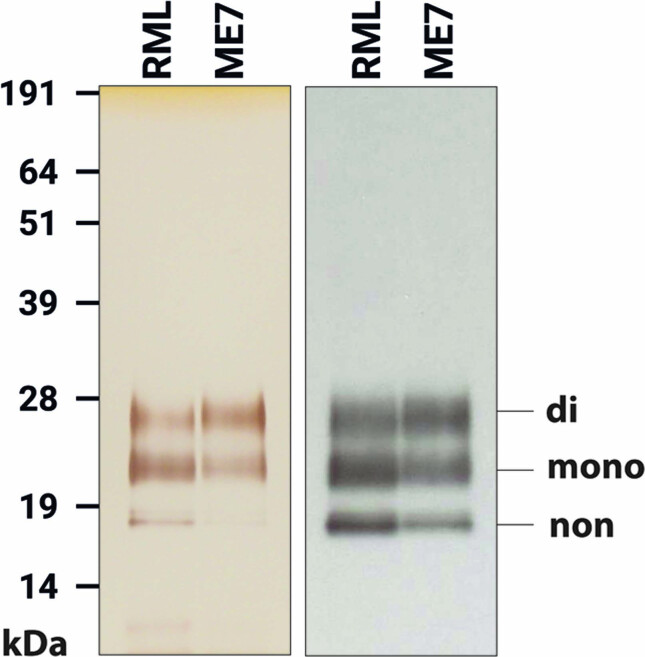
Silver-stained SDS-PAGE (left) and western blot (right) of purified ME7 and RML rods. Labelling shows the migration positions of di-, mono- and non-glycosylated PrP. The samples were prepared as described in Methods. Uncropped and unprocessed SDS-PAGE and western blot data are provided in a Source Data file. Findings are representative of ~30 purifications. Source data

ME7 prion rods were purified to ~99% purity with respect to total protein from the brain of terminally infected C57Bl/6 mice (Extended Data Fig. 1). We employed the same established method^31^ as for the analogous RML preparations^23^, which includes proteinase K (PK) treatment of crude brain homogenate and the addition of phosphotungstate (PTA) polyanions that were shown to decorate RML prion fibrils^23^ without affecting the protofilament structure^23,24^. Mass spectrometry analyses (provided in a Source Data file) showed that PK N-terminally truncates PrP monomers in ME7 rods at the same site as in RML rods^23^, leaving PrP subunits predominantly starting at residue 89 and extending to the C-terminus with intact GPI anchor. Prion infectivity of purified ME7 prions was measured using the Scrapie Cell End Point Assay^31,33–35^ (Supplementary Table 1) with titers consistent with previous findings^31,36,37^. As before^23,37^, prion rods were the only visible polymers in micrographs with the exception of occasional collagen fibers, amorphous aggregates or vesicles.

**Fig. 1:**
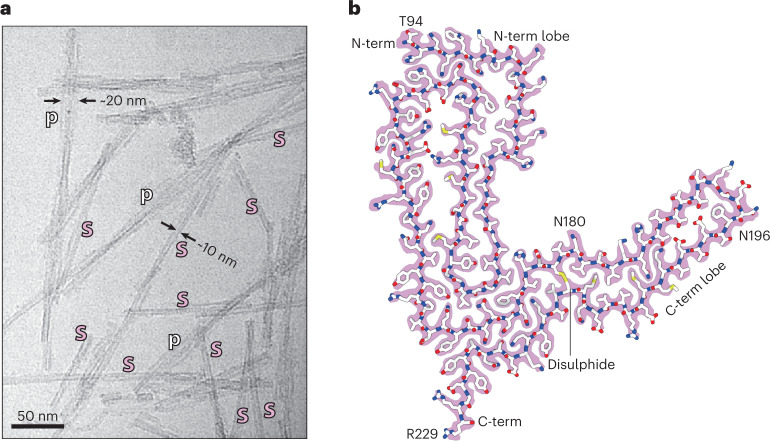
ME7 fibril morphologies and the atomic structure of their constituent PrP subunit. **a**, Representative cryo-EM image from a dataset comprising 6,370 multi-frame movies (300-kV FEI Krios G3i, K3 camera) showing examples of single ME7 protofilaments (s) alongside their paired assemblies (p) with their approximate diameters. **b**, Protein-only density of a single amyloid rung (pink) with the fitted atomic model of the mouse PrP chain shown with sticks colored by heteroatom: C, white; N, blue; O, red; S, yellow. The start (T94) and end (R229) residues of the fitted polypeptide and both N-glycosylation sites are indicated.

Among predominantly single protofilament fibrils (~10-nm apparent diameter), distinctive paired assemblies with the apparent diameter of ~20 nm were also observed in approximately 15% of the micrographs (Fig. 1a). As with the previously reported RML pairs, it remains unclear if or how PTA may influence the protofilament pairing^23^. Thus, here we focus on single ME7 protofilaments and on how they relate to the previously reported RML protofilaments^23^, whose structure is demonstrably not perturbed by PTA^24^.

We determined a 2.6-Å-resolution structure of the single ME7 protofilament and found that the fold of its constituent PrP subunit closely resembles that of the RML protofilament, including the double-hairpin N-terminal lobe and a single-hairpin DS C-terminal lobe, with four additional C-terminal residues stabilized as part of the amyloid core in the ME7 fibril (94–229) compared to the RML fibril (94–225) (Fig. 1b, Supplementary Table 2 and Supplementary Figs. 1 and 2). We, thus, appended the remaining D226–R229 residues to the previously built model^23^ and then fitted and refined that C-terminally extended model in the cryo-EM density of ME7 (Fig. 1b and Supplementary Table 2).

### General comparison of ME7 and RML protofibrils

**Fig. 2:**
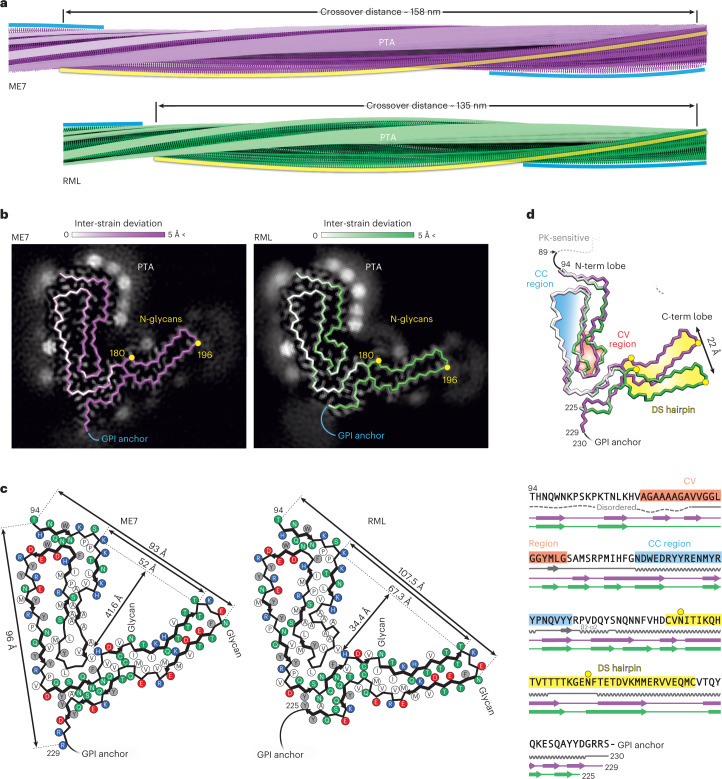
Comparison of ME7 and RML protofibrils and assignment of folding subdomains in their core. **a**, Rendered density views of the helix crossover or half-pitch (180° helical turn) distance, with annotated locations of PTA polyanions (semi-transparent white), N196 (yellow) and GPI anchor (blue). **b**, Density cross-sections with overlaid PrP backbone models colored by relative deviation based on global 3D alignment (UCSF Chimera; **d**), with annotations corresponding to **a**. C-α atoms of both N-glycosylation sites (N180 and N196) are marked with yellow circles. **c**, Diagrams of the PrP subunits with approximate dimensions of the inter-lobe grooves and the longest C-α distances in each model, measured between the indicated C-α atoms (dotted lines). Positions of amino acid side chains are shown with circles (positively charged, blue; negatively charged, red; neutral, green; hydrophobic, white; aromatic, gray) on either side of the backbone (black line). β-strands are indicated with thick black arrowheaded lines. **d**, Top, superposition of PrP backbones colored as in **b** and shaded according to the folding subdomain assignment. N-glycosylation sites are marked with yellow circles. PK-resistant core starts with residue 89. Bottom, mouse PrP sequence from the start of the amyloid core (T94) to the C-terminus (S230-GPI anchor), with color-coded folding subdomain assignment. Secondary structure annotation for PrP^C^ (gray), ME7 fibril (magenta) and RML fibril (green) is included below the sequence (α-helix, zig-zag; β-sheet, arrow; disordered, undulated dashed line). Start and end residues are numbered, and the β2-α2 region of PrP^C^ is indicated. N-glycosylation sites (N180 and N196) are marked with yellow circles.

**Extended Data Fig. 2:**
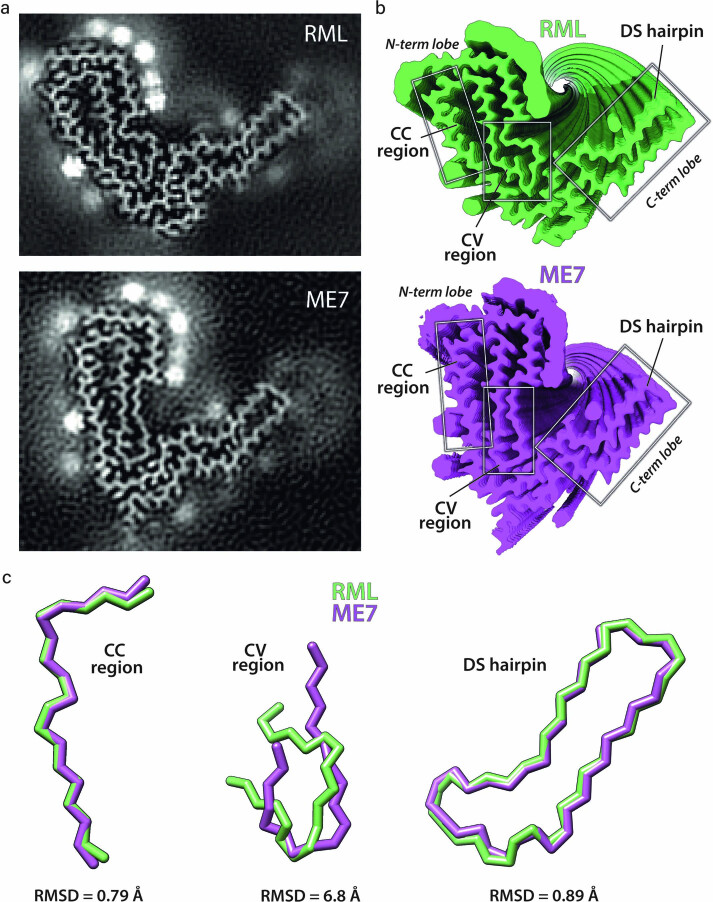
Comparisons between cryo-EM maps and atomic models of mouse prion fibrils. **a** Density cross-sections (pixel size: RML, 1.067 Å; ME7, 0.828 Å) showing the protein core and the non-protein extra densities in the final cryo-EM map. **b** Top views of rendered reconstructions with indicated common folding sub-domains. **c** backbone alignments of the common folding sub-domains and the root mean square deviations (RMSD) between all of their respective atom pairs, as calculated using UCSF Chimera^60^. CC, conformationally conserved; CV, conformationally variable; DS, disulphide-stapled.

Akin to the previously reported RML protofilaments^23,24^, the ME7 protofilaments comprise a helical PIRIBS ribbon, where each two-lobed rib or rung is formed by a single PrP chain (Fig. 2a,b and Extended Data Fig. 2a,b). The left-handed twist of the ME7 fibril is slightly slower than that of the RML fibril (approximately −0.54° versus −0.64° per rung, respectively), which results in a longer crossover distance (approximately 1,585 Å versus 1,344 Å, respectively) (Fig. 2a). Spacing between the ME7 rungs was estimated at approximately 4.79 Å, whereas, in RML, it was at approximately 4.82 Å (ref. 23), but, given the uncertainty of the pixel size calibration for each magnification (different in each dataset), these spacings can likely be considered essentially the same (~4.8 Å).

The extra (non-protein) densities surrounding the N-terminal lobe of the ME7 reconstruction are consistent with PTA cages ([PW_11_O_39_]^7−^ at pH 7.8), previously seen in corresponding locations (basic residues) around RML rods purified with the same method^23^ (Fig. 2a–c and Extended Data Fig. 2a,b). The fainter extra densities around the C-terminal lobe of the ME7 reconstruction are also consistent with those previously seen around RML protofilaments^23^ and correspond to N180-linked and N196-linked glycans of variable occupancy and the flexible GPI anchor at the C-terminus (Fig. 2b and Extended Data Fig. 2a). There are also likely at least two additional weak PTA-binding sites at residues K184 and K220 in the C-terminal lobe of ME7 and one at residue K184 in the C-terminal lobe of RML; both strains also show weak areas of unassigned density around the hydrophobic patch V202–M204 (compare Fig. 2b,c).

In each strain, the N180 glycan stems from the base of the C-terminal lobe and occupies the groove between the two lobes, which is significantly narrower and deeper in the ME7 fibril than that in the RML fibril (approximately 52 × 41.6 Å versus 67.3 × 34.4 Å, respectively) (Fig. 2b–d and Extended Data Fig. 2a,b). The N196 glycan and GPI anchor project outward in a roughly similar fashion in each fibril (Fig. 2a–c). Considering the protein backbone, the widest dimension of the ME7 rung is shorter than that of RML (96 Å versus 107.5 Å, respectively) and falls between the first (T94) and the last (R229) residue of the amyloid core (one residue away from the GPI anchor), whereas that of RML falls between the first residue of the amyloid core (also T94) and N196 (the second glycosylation site) (Fig. 2c).

### Common PrP folding subdomains in ME7 and RML protofibrils

Global three-dimensional (3D) alignment of the PrP monomer from the ME7 fibril with that from the RML fibril reveals regions of conserved and of variable conformation (Fig. 2b,d). The N-terminal lobe can be subdivided into two folding subdomains on that basis. The tip of the first hairpin contains the Ala/Gly-rich sequence (A112–G130), which adopts a modestly different conformation in the two strains (Fig. 2b–d and Extended Data Fig. 2c) and is hereafter designated the conformationally variable (CV) region. Crucially, this CV region interfaces with the C-terminal lobe, and, thus, its conformation may impact the spatial arrangement of the two lobes (Fig. 2d). Conversely, the tip and the external half of the second hairpin (N142–Y162) have closely superimposable conformations in both strains (<1-Å root mean square deviation (RMSD)) (Fig. 2b,d and Extended Data Fig. 2c); we, therefore, designate this the conformationally conserved (CC) region. The DS hairpin of the C-terminal lobe, which harbors the two glycosylation sites, is also nearly superimposable between the two strains (<1-Å RMSD), although it is markedly displaced (by 22 Å at the tip) in the ME7 structure compared to the RML structure, in line with the altered configuration of the CV region (Fig. 2d and Extended Data Fig. 2c).

### Other common structural regions in ME7 and RML protofibrils

**Fig. 3:**
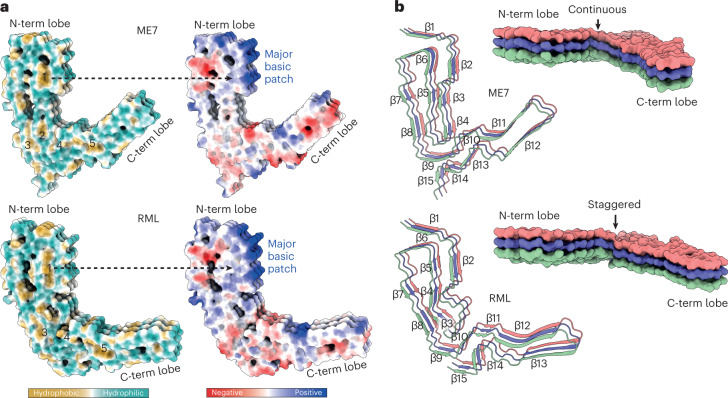
Hydrophobic and polar domains of mouse prion protofibrils and details of their PIRIBS arrangements. Models of three consecutive PrP rungs shown as: **a**, solvent-excluded surface colored by hydrophobicity and by electrostatic charge distribution (ChimeraX), and hydrophobic clusters are labeled 1–5; **b**, ribbons with secondary structures and solvent-excluded surface models colored by chain.

**Fig. 4:**
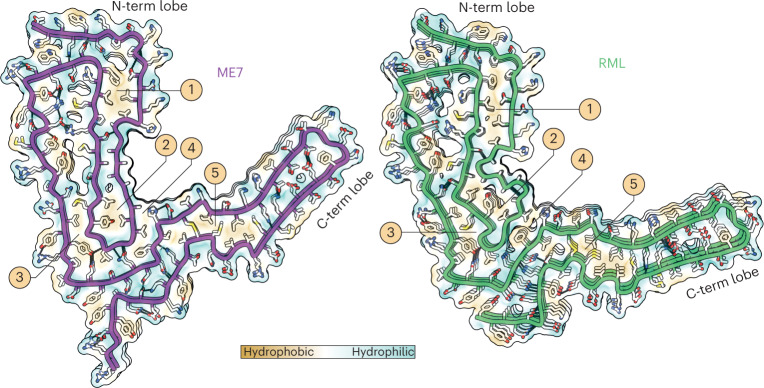
Lateral interactions stabilizing mouse prion fibrils. Depiction of polar and non-polar lateral contacts. Protein backbone is shown with cartoon (licorice) representation, amino acid side chains as white sticks colored by heteroatom (O, red; N, blue; S, yellow) and selected hydrogen bonds as black lines. The hydrophobic patches are numbered 1–5.

K100–H110 region forms a major basic patch in the N-terminal lobe of both fibrils and faces the N180 glycan in the groove of the fibril (Figs. 2b,c and 3a,b). This basic patch appears to be firmly stabilized by the first of the five major intra-chain hydrophobic clusters (mainly P101, P104, L108, V111, P136, I138 and F140), in both ME7 and RML fibrils (Figs. 2b,3a and 4). The second and third major hydrophobic clusters (mainly V121, Y126, L128 and L123, M127, V160, Y162 and P164, respectively) appear to stabilize variable configurations of the CV region within the N-terminal lobe, whereas the fourth major hydrophobic cluster provides the third hydrophobic anchor point for the CV region at the inter-lobe interface in both protofibrils (Figs. 2c,3a and 4). This hydrophobic interface involves the N-terminal lobe’s residue V120 and the C-terminal lobe’s residues F174 and H176 in both RML and ME7 folds and, additionally, N-terminal lobe’s residue A119 in the ME7 fold only (Figs. 2c and 4). Finally, the fifth major hydrophobic cluster (I181, I183, M205, V208, V209 and M212) precedes and encompasses the base of the DS hairpin (spanning both sides of the disulphide bond), likely conferring rigidity to the DS hairpin fold in both structures (Figs. 2c,3a and 4). Of note, the interaction of V175 with V214 in the ME7 protofibril is replaced by a less favorable interaction with T215 in RML (Fig. 2c).

### Distinct PIRIBS arrangement in ME7 and RML protofibrils

There are 15 inter-chain β-sheets (or PIRIB-sheets) in ME7 and RML protofibril structures, but their arrangement is not identical (Figs. 2c,d and 3b). The largest variation in the β-sheet distribution is seen in the CV region, and the highest conservation of that is seen in the CC region (β-strands 6–8) and the DS hairpin (β-strands 10–12 in ME7 and 11–13 in RML) (Figs. 2c,d and 3b). Intervening regions also show variations in the PIRIBS arrangement (Figs. 2c,d and 3b), but the first two β-strands that accompany the major basic patch are conserved (Figs. 2c,3a,b and 4). The PIRIBS architecture defines the main longitudinal polar inter-chain interactions, the inter-rung hydrogen bonds, but, besides the hydrophobic interactions described earlier, there are also lateral intra-chain and inter-chain hydrogen bonds that stabilize both folds (Fig. 4).

Notably, all types of lateral associations between neighboring strands are not exactly co-planar but staggered by ~half-rung distance along the helical axis (that is, running between rungs). The most pronounced stagger (by nearly one rung) is seen at the inter-lobe interface of the RML protofibril (that is, the N-terminal lobe of the rung *i* interacts with the C-terminal lobe of mainly the rung *i* + 1). In the ME7 protofibril, this interface is more continuous, showing the standard ~half-rung stagger (that is, the N-terminal lobe of the rung *i* interacts equally with C-terminal lobes of the rungs *i* and *i* + 1) (Fig. 3b).

## Discussion

**Fig. 5:**
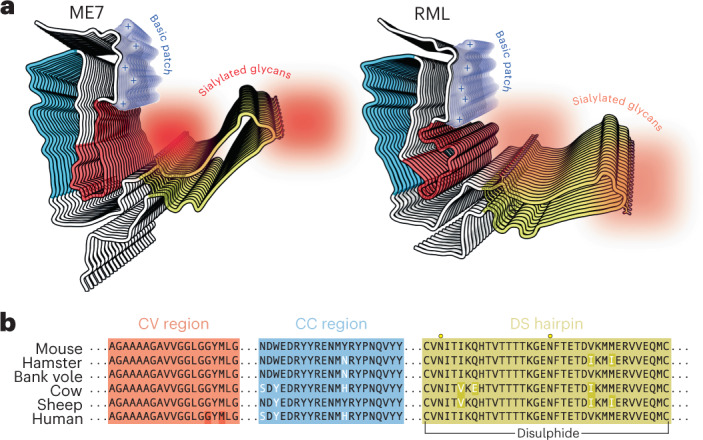
Summary of structural differences between ME7 and RML prion protofibril structures. **a**, Licorice backbone models (ChimeraX) of ME7 and RML protofibrils colored according to PrP^Sc^ folding subdomains (blue, CC region; red, CV region; yellow, DS hairpin; white, intervening regions). Positive charge of the major basic patch (transparent surface) is indicated with + signs, and the sialoglycan occupancy corresponds to the intensity of red shading. The N-glycosylation sites (N180 and N196 side chains) and the disulphide bond are shown as sticks and colored by heteroatom (C, yellow; N, blue; O, red; S, yellow). **b**, Alignment of PrP sequences from different mammals focused on the PrP^Sc^ folding subdomains. Human residues 127 and 129 in the CV region (highlighted with dark red) are polymorphic (G/V and M/V, respectively).

**Extended Data Fig. 3:**
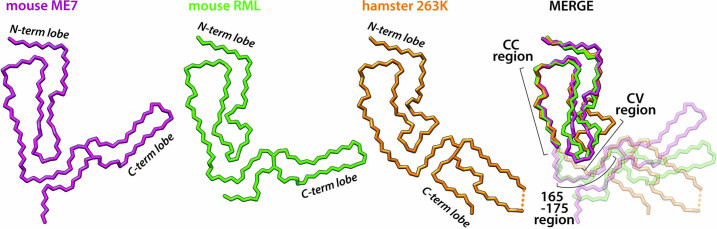
Comparison of rodent prion structures. Backbones of the three structures are aligned on the conformationally conserved (CC) region in the N-terminal lobe (MERGE, dimmed beyond the CC region). The C-terminal lobe of mouse prion strains has divergent orientations in line with differences in the N-terminal lobe’s conformationally variable (CV) region that interfaces with the C-terminal lobe. The C-terminal lobe of the hamster strain diverges further from the mouse strains due to additional differences in its primary structure (PrP sequence). The 165-176 region, which corresponds to the β2-α2 loop in PrP^C^, is part of the inter-lobe interface in all three rodent prion fibril structures.

The infectious RML and ME7 mouse prion protofibril structures compared here show a remarkably similar modular architecture, comprising two conformationally congruent modules (the CC region in the N-terminal lobe and the DS hairpin in the C-terminal lobe) connected by a CV module (the CV region) at the core of the assembly (Fig. 5). A similar overall architecture is seen in the hamster 263K protofibril^22–24^ (Extended Data Fig. 3). Comparison of the three folds suggests that the low-complexity (Gly/Ala-rich) region of PrP (residues 112–130) (Fig. 5b) that comprises the CV region is likely amenable to a greater range of misfolded configurations, whereas other more complex regions of sequence that comprise the CC region and DS hairpin (Fig. 5b) may be more conformationally restricted (Extended Data Fig. 3). Of note, in all three folds, the inter-lobe interface involves interaction of residues of the CV region with residues that comprise the β2-α2 loop in the normal PrP^C^ fold (residues 165–175)^38–40^ (Fig. 2d and Extended Data Fig. 3). Variation of amino acid sequence within the β2-α2 loop has substantial effects on numerous interspecies prion transmission barriers^41–45^, and the CV region contains the 127 G/V and 129 M/V polymorphisms of human PrP that profoundly affect prion disease susceptibility, phenotype and strain selection^2–4,29,46^. Thus the inter-lobe interface appears to be a critical structural determinant of conformational selection linking strains and prion transmission barriers. Although structures of protofibrils from other prion strain/host combinations are clearly required to inform on the generalizability of these new findings, these data now suggest an initial structural framework underpinning prion strain diversity in mammals. In this regard, the new cryo-EM structures coupled with the general similarity of PrP 27–30 truncated PrP^Sc^ banding patterns seen across multiple human and animal prion strains firmly point to a commonality of PIRIBS-based fibrillar architectures.

Notably, the K100–H110 sequence is part of the disordered N-terminal domain in PrP^C^ (refs. 38–40) (Fig. 2d), but, in both mouse (ME7 and RML) protofibrils and in the hamster 263K protofibril, that amino acid stretch forms a common physicochemical motif, the major basic patch, around the second β-strand of the N-terminal lobe (compare Figs. 2c,3a,b and 5a and Extended Data Fig. 3)^22–24^. This basic patch faces the N180 glycan in both RML and ME7 mouse protofibrils and also in the hamster protofibril^22–24^ and may, therefore, interact with sialylated N180 glycans. However, PTA polyanions used in our ex vivo prion fibril preparations clearly line the major basic patch of both mouse prion protofibrils (Fig. 2b) and, in turn, change its charge from positive to negative. This change does not unify the geometry of ME7 and RML protofibrils, suggesting that the strain-specific topology of the N-terminal and C-terminal lobes in mature prion strains is locked by the PIRIBS architecture alone and that the folds do not depend on long-range sialylated glycan–protein interactions. This deduction is firmly supported by the finding that the wild-type RML fibril fold can be stably propagated using mouse PrP lacking post-translational modifications^23,24^ and the demonstration that RML and ME7 prion fibrils purified with PTA faithfully retain their strain-specific transmission properties in mice^31^. The fact that PTA decoration of RML and ME7 fibrils appears to have no significant impact on strain-specific biological activity directly attests to the biological relevance of the fibril folds that we report. Notably, although maintenance of the strain-specific RML and ME7 fibril folds does not appear to rely upon long-range sialylated glycan–protein interactions, the incorporation of particular PrP glycoforms into nascent prion fibrils at the inception of the strain may critically determine the configuration of the fold^23^.

The protease-resistant cores and PrP glycoform ratios of purified RML, ME7 and 263K prion fibrils are congruent with the PrP 27–30 truncated PrP^Sc^ banding patterns seen on western blots of PK-digested crude brain homogenates^22,23,31^. All three strains have distinct PrP glycoform ratios, with both the 263K and ME7 strains having a greater proportion of di-glycosylated PrP than the RML strain, which contains relatively more mono-glycosylated and non-glycosylated PrP chains. As stacking of solely di-glycosylated PrP chains into a PIRIBS architecture does not appear energetically prohibitive^47^, and comparison of the cross-sections of the RML, ME7 and 263K single protofilaments (Extended Data Fig. 3) does not suggest an obvious steric basis for glycoform selection, it remains unclear how the single protofibril architectures might dictate glycoform composition. On this basis, and as previously proposed^23^, we suggest that paired fibril assemblies may have a critical role in determining prion strain-specific PrP glycoform signatures. These pairs currently represent a minor subpopulation (10–15%) of assemblies observed in our RML and ME7 fibril preparations. However, we do not know how different prion purification methods may distort the true (in vivo) content of the pairs. For example, PTA cages are found adjacent to various pairing interfaces and may be disruptive^23^. Whether relatively harsh purification conditions used by others—for example, 1.7 M NaCl (ref. 22)—may also be disruptive remains to be established. Structural investigation of the pairs purified without PTA is currently ongoing and may shed more light on how prion strain-specific glycoform ratios are generated.

## Methods

### Research governance

Prion purification, cell-based prion bioassay and preparation of cryo-EM grids was conducted at University College London (UCL) in microbiological containment level 3 or level 2 facilities with strict adherence to safety protocols. Work with infectious prion samples at Birkbeck College London was performed using dedicated sample holders and equipment with strict adherence to safety procedures and local risk assessment. Prion samples were transported between laboratories in packaging conforming to UN 3373 Biological Substance, Category B specifications. Frozen brains from mice with clinical prion disease were used to generate purified prion samples. These brain samples were generated by us as part of a previous study^31^ in which work with animals was performed in accordance with licenses approved and granted by the UK Home Office (project licenses 70/6454 and 70/7274) and conformed to UCL institutional and ARRIVE guidelines. All experimental protocols were approved by the local research ethics committee of UCL Queen Square Institute of Neurology/National Hospital for Neurology and Neurosurgery.

### Preparation of purified ME7 prion rods

Prion-infected brain homogenate was prepared by homogenizing 30 brains from female C57Bl/6 mice terminally infected with the ME7 prion strain in Dulbecco’s PBS (D-PBS) lacking Ca^2+^ or Mg^2+^ ions (Gibco) to produce a pool of 130 ml of 10% (w/v) ME7 brain homogenate (designated I21487) using established methods^31^. Purification of ME7 prion rods was performed using the protocol of Wenborn et al.^31^ with the exception first implemented in Manka et al.^23^ that initial protease digestion was performed using PK in the place of pronase E. Accordingly, 200-µl aliquots of 10% (w/v) ME7 brain homogenate were dispensed into standard 1.5-ml microfuge tubes with screw cap and rubber O-ring. Typically, 12 tubes were processed at a time. Samples were treated with 2 µl of 5 mg ml^−1^ PK prepared in water (to give 50 µg ml^−1^ final protease in the sample) and incubated for 30 minutes at 37 °C with constant agitation, after which digestion was terminated by the addition of 4.1 µl of 100 mM 4-(2-aminoethyl) benzenesulfonyl fluoride hydrochloride (AEBSF) to give 2 mM final concentration in the sample. Then, 206 µl of 4% (w/v) sarkosyl (Calbiochem) in D-PBS and 0.83 µl of benzonase (purity 1; 25,000 U ml^−1^) were added to give final concentrations in the sample of 2% (w/v) and 50 U ml^−1^, respectively. After incubation for 10 minutes at 37 °C, 33.5 µl of 4% (w/v) sodium phosphotungstate (NaPTA) prepared in water pH 7.4 was added to give a final concentration of 0.3% (w/v) in the sample. After incubation for 30 minutes at 37 °C, the samples were adjusted (and thoroughly mixed) with 705.3 µl of 60% (w/v) iodixanol and 57.2 µl of 4% (w/v) NaPTA prepared in water pH 7.4, to give final concentrations in the sample of 35% (w/v) and 0.3% (w/v), respectively. After centrifugation for 90 minutes at 16,100*g*, the sample separates into an insoluble pellet fraction (P1), a clarified supernatant (SN1) and a buoyant, partially flocculated, surface layer (SL). One milliliter of SN1 was carefully isolated from each tube, taking extreme care to avoid cross-contamination with either P1 or SL. SN1 was filtered using an Ultrafree HV microcentrifuge filtration unit (0.45-µm pore size Durapore membrane; Millipore, UFC30HV00). This was accomplished by loading 500-µl aliquots of SN1 and centrifugation at 12,000*g* for 30 seconds using one filtration unit per milliliter of SN1. Then, 480-µl aliquots of filtered SN1 were transferred to new 1.5-ml microfuge tubes and thoroughly mixed with an equal volume of 2% (w/v) sarkosyl in D-PBS containing 0.3% (w/v) NaPTA pH 7.4 and incubated for 10 minutes at 37 °C. Samples were then centrifuged for 90 minutes at 16,100*g* to generate an insoluble pellet fraction (P2) and a clarified supernatant (SN2). SN2 was carefully removed and discarded, after which each P2 pellet was resuspended in 10 µl of 5 mM sodium phosphate buffer pH 7.4 containing 0.3% (w/v) NaPTA and 0.1% (w/v) sarkosyl. To avoid unnecessary aggregation of the purified rods arising from repeated rounds of centrifugation, the final two wash steps detailed in Wenborn et al.^31^ were replaced with a single wash. Resuspended P2 pellets were pooled and mixed with 1.0 ml of 5 mM sodium phosphate buffer pH 7.4 containing 0.3% (w/v) NaPTA and 0.1% (w/v) sarkosyl, and samples were centrifuged at 16,100*g* for 30 minutes to generate a clarified supernatant (SN3) and an insoluble pellet fraction (P3). SN3 was carefully removed and discarded, and final P3 samples were typically resuspended to a concentration of 150–200× relative to the starting volume of 10% (w/v) brain homogenate from which they were derived, before loading onto EM grids (see below).

Prion infectivity of brain homogenates or purified samples was measured using the Scrapie Cell End Point Assay^31,33–35^ using LD9 cells (an established cell line, which was a gift from Charles Weissmann and originally derived from murine L929 fibroblasts supplied by the American Type Culture Collection^34^). Every experiment included concomitant assay of a serial dilution of RML prions of known prion titer determined from rodent bioassay. Ten percent (w/v) RML brain homogenate I6200 was used as the standard and reported a prion titer of 10^7.3 + 0.5^ (mean + s.d.) intracerebral LD_50_ units per milliliter when endpoint titrated six times in Tg20 mice that overexpress mouse PrP on a *Prnp*^o/o^ background^31^. PrP concentrations in purified samples were measured by ELISA^31^.

### SDS-PAGE, silver staining and western blotting

Samples were prepared for SDS-PAGE using NuPAGE 4× LDS buffer and 10× Reducing Agent (Thermo Fisher Scientific) according to the manufacturer’s instructions, followed by immediate transfer to a 100 °C heating block for 10 minutes. Electrophoresis was performed on NuPAGE 12% Bis-Tris protein gels (Thermo Fisher Scientific), run for 60 minutes at 200 V, before electroblotting to Immobilon P membrane (Millipore) for 16 hours at 15 V. Membranes were blocked in 1× PBS (prepared from 10× concentrate; VWR International) containing 0.05% (v/v) Tween 20 (PBST) and 5% (w/v) non-fat dried skimmed milk powder and then probed with 0.2 μg ml^−1^ of anti-PrP monoclonal antibody ICSM35 (D-Gen Ltd.) in PBST for at least 1 hour. After washing (1 hour with PBST), the membranes were probed with a 1:10,000 dilution of alkaline-phosphatase-conjugated goat anti-mouse IgG secondary antibody (Sigma-Aldrich, A2179) in PBST. After washing (1 hour with PBST and 2 × 5 minutes with 20 mM Tris pH 9.8 containing 1 mM MgCl_2_), blots were incubated for 5 minutes in chemiluminescent substrate (CDP-Star, Tropix Inc.) and visualized on Biomax MR film (Carestream). SDS-PAGE gels (prepared as above) were silver stained using the Pierce Silver Stain Kit (Thermo Fisher Scientific) according to the manufacturer’s instructions. Gels were photographed on a light box using a Nikon Coolpix P6000 digital camera. Typical sample loadings for western blotting or silver staining correspond to purified material derived from 10 µl or 100 μl of 10% (w/v) prion-infected brain homogenate per lane, respectively. The SDS-PAGE and western blot data generated in this study are provided in a Source Data file.

### ME7 sample preparation for cryo-EM

ME7 prion rods purified from 2.4 ml of 10% (w/v) ME7-infected brain homogenate were resuspended from the P3 pellet (see above) in 10–20 μl of 5 mM sodium phosphate buffer pH 7.4 containing 0.1% (w/v) sarkosyl, and 4 μl of the suspension was applied directly to a glow-discharged C-flat Holey Carbon CF-2/2-4C Cu 400 mesh cryo-EM grid (Electron Microscopy Sciences) or Quantifoil R2/2 Cu 300 mesh grids in the chamber of the Leica GP2 plunging robot. The chamber was set to 20 °C and 40% humidity. After 10-second incubation, the grids were blotted for 3 seconds (with an additional 2-mm push) and plunge-frozen in liquid ethane maintained at −183 °C.

### Cryo-EM data collection

Cryo-micrographs were acquired at Birkbeck College London on a 300-kV Krios G3i microscope (FEI/Thermo Fisher Scientific) with a post-GIF (20-eV slit) K3 detector (Gatan) operated in super-resolution bin 2× mode at 105,000 nominal magnification. The final (post-binning) magnified pixel size was 0.828 Å. The dose rate was ~19.9 e-/Å^2^/s during 2.5-second exposures, resulting in a total dose of ~49.75 e-/Å^2^ on the specimen. The exposures were collected automatically at five shots per grid hole, with fast acquisition (up to ~370 images per hour), using the EPU 2 software (FEI/Thermo Fisher Scientific), at defocus ranging from 2.4 μm to 0.9 μm and fractionated into 50 movie frames.

### Cryo-EM image processing and 3D reconstruction

All image processing except filament picking was done within the RELION 4.0-beta framework^48^. We used RELION’s implementation of the MotionCor2 algorithm to align movie frames. The contrast transfer function (CTF) parameters were estimated with CTFFIND4 (ref. 49). We then trained the deep learning package crYOLO^50^ to pick ME7 filaments using 100 example micrographs, as previously reported^23^. We imported the coordinates into RELION and extracted images of prion rod segments with different box sizes (ranging from 1,024 to 384 pixels) to perform reference-free two-dimensional (2D) classifications. Optimal 2D class averages and segments were selected for further processing and used to de novo generate an initial 3D reference with the relion_helix_inimodel2d program^51^, using an estimated rise of 4.79 Å and helical twist according to the observed crossover distances of the filaments in the 2D class averages. After 3D classification and 3D auto-refinement, we obtained a 3D reconstruction of the ME7 protofibril at 2.9-Å resolution. Subsequent Bayesian polishing^52^ and CTF refinement^53^ were performed to further improve the resolution of the reconstruction to 2.6 Å, according to the 0.143 Fourier shell correlation (FSC) cutoff criterion (Supplementary Fig. 2). The final 3D map was sharpened with a generous, soft-edged solvent mask at 10% of the height of the box using the computed B-factor value of −26.75 Å^2^. The sharpened map was used for the subsequent atomic model building and refinement. The absolute hand of the helical twist was determined directly from the map through resolved densities of the carbonyl oxygen atoms of the polypeptide backbone^51^. The local resolution calculation was performed by LocRes in RELION 4.0-beta with solvent mask over the entire map.

### Atomic model building and refinement

A single subunit repeat was extracted from the cryo-EM map of the ME7 protofibril in UCSF Chimera^54^. A single PrP chain from the previously determined atomic model of the RML fibril structure (Protein Data Bank (PDB) ID: 7QIG)^23^ was C-terminally extended by addition of D226–R229 residues and fitted to the extracted ME7 density in Coot^55^. The initially fitted atomic model was then copied and fitted into three consecutive subunits in the ME7 map, and the map was zoned around the atomic coordinates in UCSF Chimera^54^. The three-rung map and model were placed in a new unit cell with P1 space group for subsequent model refinement using default settings in phenix.real_space_refine^56^ and REFMAC5 (ref. 57). Model geometry was evaluated using MolProbity^58^ (http://molprobity.biochem.duke.edu/) after each round of refinement, and problematic or poorly fitting regions in the model were manually adjusted using Coot^55^ and ISOLDE^59^ (within ChimeraX^60^). This process was repeated until a satisfactory level of model:map agreement with acceptable model stereochemistry was achieved (Supplementary Table 2).

### Structure analyses and presentation

Analyses and visualizations of the cryo-EM density map and the models compared in this study were done using UCSF Chimera^54^ and ChimeraX^60^.

### Determination of N-terminal PK cleavage sites by mass spectrometry

N-terminal PK cleavage sites in PrP subunits of ME7 fibrils were determined by targeted derivatization of α-amino groups and subsequent analysis by mass spectrometry as done previously for RML fibrils^23^. In brief, purified ME7 fibrils were electrophoresed in NuPAGE 12% Bis-Tris mini protein gels (Thermo Fisher Scientific), after which gel sections spanning all three PrP glycoforms were excised. Gel pieces were reduced with 100 µM tris(2-carboxyethyl)phosphine and alkylated with 200 µm of iodoacetamide before N-terminal labeling with 6 mM N-succinimidyloxycarbonylmethyl tris(2,4,6-trimethoxyphenyl)phosphonium bromide (TMPP-Ac-OSu) (Sigma-Aldrich) for 1 hour at 22 °C in 100 mM HEPES buffer pH 8.2. After washing, gel pieces were digested overnight with trypsin at a working concentration of 2.5 µg ml^−1^. Tryptic digest peptides were recovered from the gel and analyzed by liquid chromatography–mass spectrometry, using an Acquity I-Class UPLC system coupled to a Xevo G2-XS Q-ToF mass spectrometer (Waters). Data were collected in MSe acquisition mode using concurrent low-collision and high-collision energy functions with 5 V and 15–45 V of collision energy, respectively. ProteinLynx Global Server 3.0.3 (Waters) and a species-specific reference proteome (UniProt UP000000589, *Mus musculus*) were used to assign peptide sequences optionally allowing for N-terminal amino-group derivatization by TMPP (+572.1811 Da). For each TMPP-labeled peptide, extracted ion chromatograms were generated, and their relative abundance was determined from their respective peak areas. These data are provided in a Source Data file.

### Statistics and reproducibility

Purification of ME7 prions was successfully replicated ~30 times while optimizing sample concentrations for cryo-freezing using a 120-kV Talos microscope (FEI/Thermo Fisher Scientific). Eight cryo-EM grids containing material from four independent prion purifications were used for data collection in the Krios G3i microscope. Representative images of prion rods in ice were selected from a dataset comprising 6,370 multi-frame movies.

### Reporting summary

Further information on research design is available in the Nature Portfolio Reporting Summary linked to this article.

## Online content

Any methods, additional references, Nature Portfolio reporting summaries, source data, extended data, supplementary information, acknowledgements, peer review information; details of author contributions and competing interests; and statements of data and code availability are available at 10.1038/s41589-022-01229-7.

## Supplementary information

Supplementary Information Supplementary Tables 1 and 2, Supplementary Figs. 1 and 2 and Supplementary References.

Reporting Summary

## Data Availability

The ME7 cryo-EM density map was deposited into the Electron Microscopy Data Bank (EMDB) (https://www.ebi.ac.uk/pdbe/emdb) under accession number EMD-15043 (Infectious mouse-adapted ME7 scrapie prion fibril purified from terminally-infected mouse brains). The corresponding atomic coordinates were deposited in the Protein Data Bank (PDB) (https://www.rcsb.org) under PDB ID 8A00. The RML 3D cryo-EM density map was accessed from the EMDB under accession number EMD-13989 (Infectious mouse-adapted RML scrapie prion fibril purified from terminally-infected mouse brains). The corresponding atomic coordinates were accessed from the PDB under PDB ID 7QIG. The atomic coordinates of the hamster 263K prion fibril (infectious mammalian prion fibril: 263K scrapie) were accessed from the PDB under ID 7LNA. UniProt UP000000589, *Mus musculus*, was used as the reference proteome for mass spectrometry. Uncropped and unprocessed SDS-PAGE and western blot data and mass spectrometry data generated in this study are provided in the Source Data files. Source data are provided with this paper.

## Source data

Source Data Extended Data Fig. 1 Unprocessed western blot and gel.
Source Data Extended Data Fig. 1 Mass spectrometry data.
