# Supplementary material for: A structural basis for prion strain diversity
Source: Nat Chem Biol. 2023 Jan 16;19(5):607–13. doi: 10.1038/s41589-022-01229-7 (PMC10154210; doi:10.1038/s41589-022-01229-7)
Supplement: Supplementary file 1 — Supplementary Tables 1 and 2, Supplementary Figs. 1 and 2 and Supplementary References. [file 41589_2022_1229_MOESM1_ESM.pdf]

nature chemical biology

Article https://doi.org/10.1038/s41589-022-01229-7

# A structural basis for prion strain diversity

In the format provided by the authors and unedited

## Table of Contents:

Supplementary Table 1. Determination of prion infectivity titre in cell culture

Supplementary Table 2. Cryo-EM data collection, refinement and validation statistics

Supplementary Figure 1. 2D classification of ME7 prion fibrils

Supplementary Figure 2. Quality of the ME7 map and model

Supplementary references

## Supplementary Table 1. Determination of prion infectivity titre in cell culture

| Dilution | Positive Wells ($w/w_0$) | | | Percentage Positivity | | |
|---|---|---|---|---|---|---|
| | RML BH I6200* | ME7 BH I21487 | Purified ME7 (1X)† | RML BH I6200* | ME7 BH I21487 | Purified ME7 (1X)† |
| $1.10^{-4}$ | 24 / 24 | 24 / 24 | 24 / 24 | 100.0% | 100.0% | 100.0% |
| $3.10^{-5}$ | 24 / 24 | 24 / 24 | 24 / 24 | 100.0% | 100.0% | 100.0% |
| $1.10^{-5}$ | 20 / 24 | 24 / 24 | 15 / 24 | 83.3% | 100.0% | 62.5% |
| $3.10^{-6}$ | 5 / 24 | 16 / 24 | 5 / 24 | 20.8% | 66.7% | 20.8% |
| $1.10^{-6}$ | 2 / 24 | 9 / 24 | 1 / 24 | 8.3% | 37.5% | 4.2% |
| $3.10^{-7}$ | 0 / 24 | 2 / 24 | 0 / 24 | 0.0% | 8.3% | 0.0% |
| $1.10^{-7}$ | 0 / 24 | 1 / 24 | 0 / 24 | 0.0% | 4.2% | 0.0% |
| NI | 0 / 24 | 0 / 24 | 0 / 25 | 0.0% | 0.0% | 0.0% |

| | | | |
|---|---|---|---|
| **log TCIU $ml^{-1}$ $** | **5.8** | **6.1** | **5.5** |

**Measurement of total protein and PrP content:**

| | | | |
|---|---|---|---|
| **Total protein (mg $ml^{-1}$)** | **10.0** | **10.0** | **0.0027** |
| **PrP content (µg $ml^{-1}$)** | **5.4** | **11.0** | **2.7** |

**Calculation of specific infectivity:**

| | | | |
|---|---|---|---|
| **log TCIU $mg^{-1}$ protein** | **4.8** | **5.1** | **8.1** |
| **log TCIU $mg^{-1}$ PrP** | **8.1** | **8.1** | **8.1** |

($w/w_0$), the number of prion infected wells (w) versus the total number of inoculated wells ($w_0$); BH, brain homogenate; TCIU, tissue culture infectious units; NI, non-prion inoculated

* Prion titre of $10^{7.3 + 0.5}$ (mean + s.d., n=6) intracerebral $LD_{50}$ units/ml in Tg20 mice[1]
† Purified material was resuspended to the same volume as the 10% (w/v) BH from which it was derived
$ TCIU calculated according to the Poisson distribution[2]

**Supplementary Table 2. Cryo-EM data collection, refinement and validation statistics**

| | ME7 (EMD-15043) (PDB 8A00) |
|---|---|
| **Data collection and processing** | |
| Magnification | 105,000 x |
| Voltage (kV) | 300 |
| Electron exposure (e–/Å$^2$) | 49.4 |
| Defocus range (μm) | from -2.4 to -0.9 |
| Pixel size (Å) | 0.828 |
| Symmetry imposed | C1 |
| Initial particle images (no.) | 223,065 |
| Final particle images (no.) | 40,239 |
| Map resolution (Å) | 2.6 |
| FSC threshold | 0.143 |
| Map resolution range (Å) | 2.6-3 |
| | |
| **Refinement** | |
| Initial model used (PDB code) | 7QIG |
| Model resolution (Å) | 2.6 |
| FSC threshold | 0.143 |
| Model resolution range (Å) | 121.7 - 2.6 |
| Map sharpening *B* factor (Å$^2$) | -26.75 |
| **Model composition** | |
| Non-hydrogen atoms | 6456 |
| Protein residues | 408 |
| Ligands | none |
| ***B* factors (Å$^2$)** | |
| Protein | 24.18-67.46 |
| **r.m.s. deviations** | |
| Bond lengths (Å) | 0.002 |
| Bond angles (°) | 0.580 |
| **Validation** | |
| MolProbity score | 1.49 |
| Clashscore | 2.79 |
| Poor rotamers (%) | 0 |
| **Ramachandran plot** | |
| Favored (%) | 93.53 |
| Allowed (%) | 6.47 |
| Disallowed (%) | 0 |

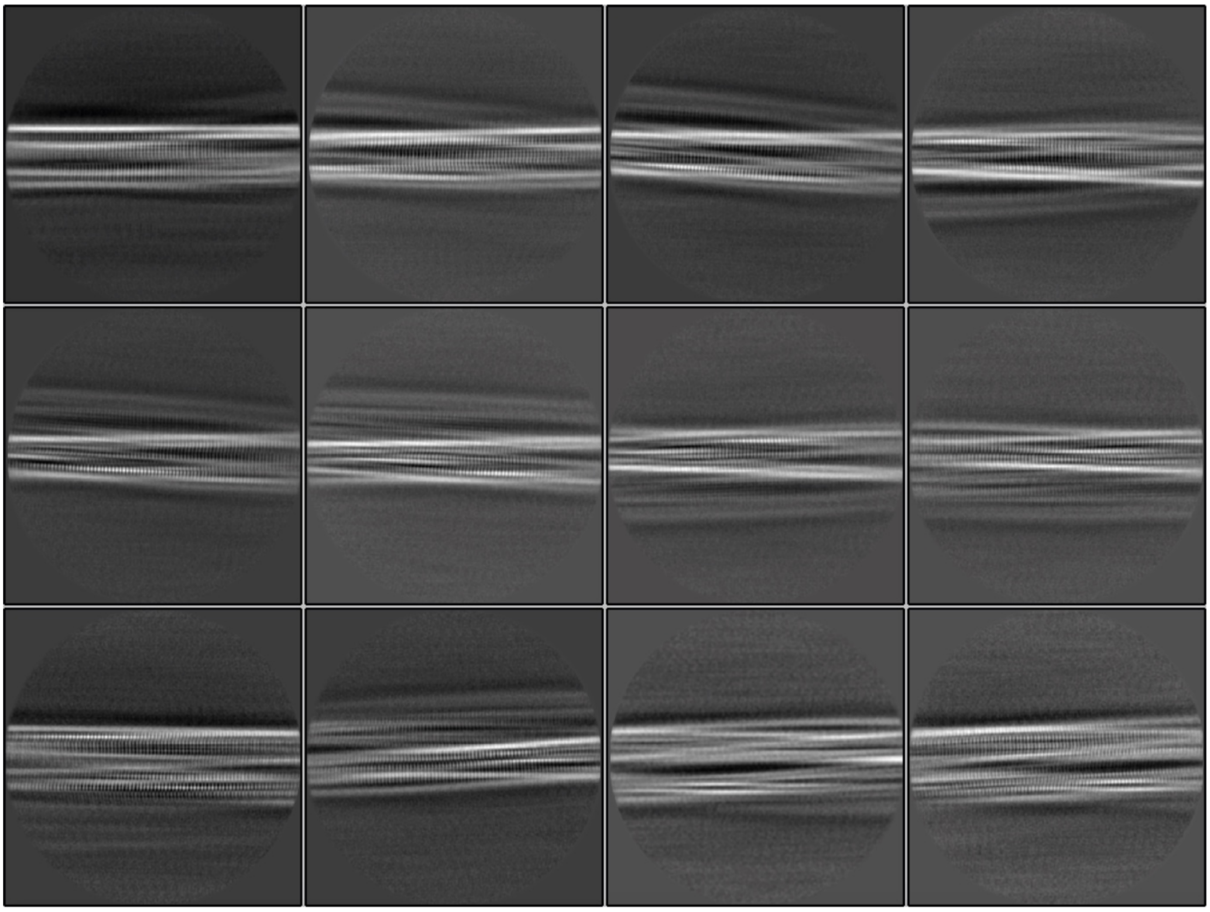

**Supplementary Figure 1. 2D classification of ME7 prion fibrils.** Gallery of representative 2D classes (box size: 397.5 x 397.5 Å).

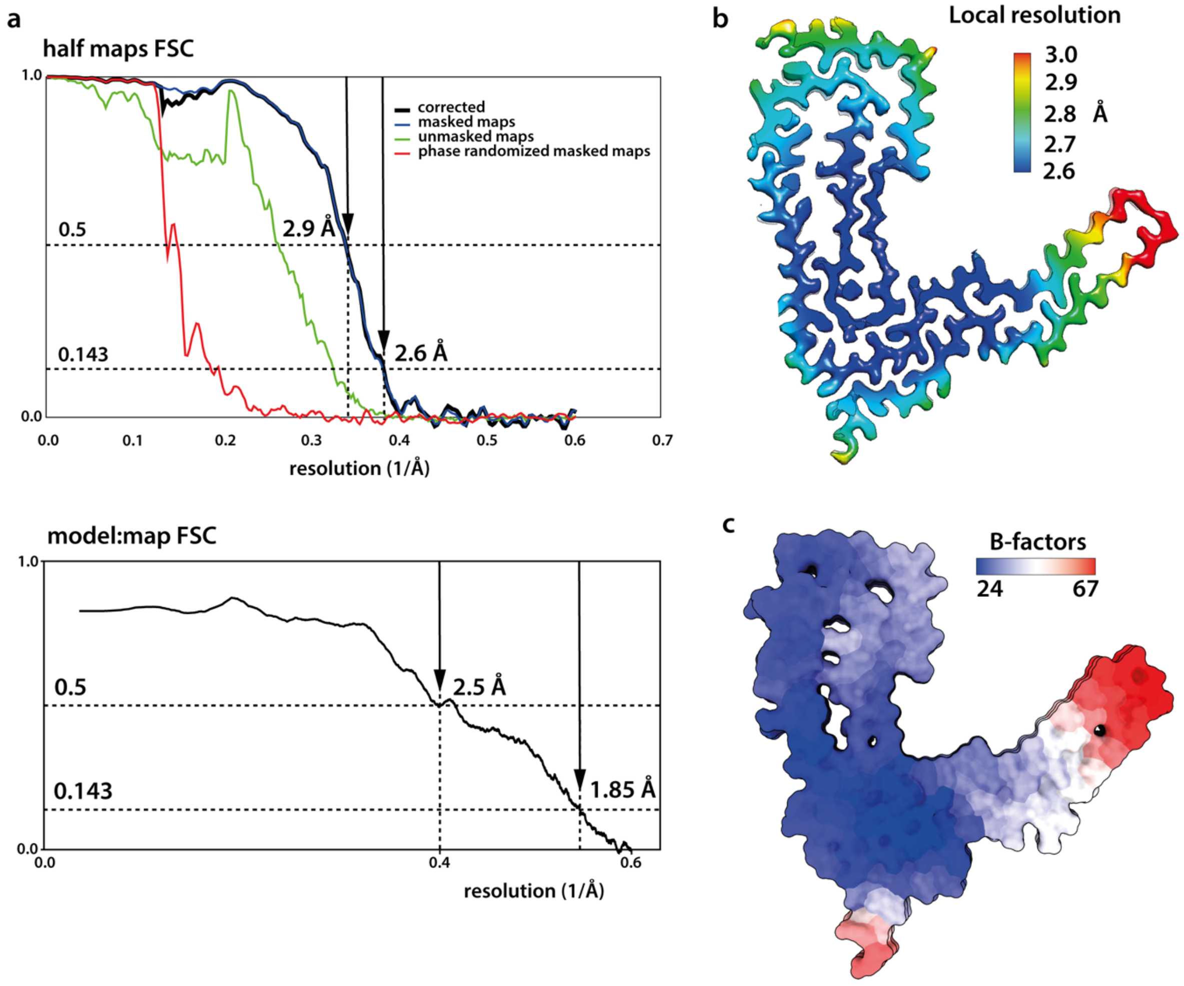


**Supplementary Figure 2. Quality of the ME7 map and model. a** Top, Fourier Shell Correlation (FSC) plots for independent half-reconstructions, determined in Relion 4.0-beta. The final plot (black line) is corrected for overfitting with high-resolution noise substitution. Bottom, FSC plot for the model:map fit. **b** Local resolution of the protein density calculated with Relion 4.0-beta LocRes. **c** Atomic B-factor values colour-coded on the solvent-excluded model surface.

**Supplementary references**

1 Wenborn, A. et al. A novel and rapid method for obtaining high titre intact prion strains from mammalian brain. *Sci Rep* **5**, 10062 (2015).

2 Edgeworth, J. A., Jackson, G. S., Clarke, A. R., Weissmann, C., & Collinge, J. Highly sensitive, quantitative cell-based assay for prions adsorbed to solid surfaces. *Proc Natl Acad Sci USA* **106**, 3479-3483 (2009).
