# Supplementary material for: A structural basis for prion strain diversity
Source: Nat Chem Biol. 2023 Jan 16;19(5):607–13. doi: 10.1038/s41589-022-01229-7 (PMC10154210; doi:10.1038/s41589-022-01229-7)

Source Data for Extended Data Fig. 1

A structural basis for prion strain diversity

Szymon W. Manka, Adam Wenborn, Jemma Betts, Susan Joiner, Helen R. Saibil, John Collinge and Jonathan D.F. Wadsworth

Source data

Images of original silver-stained SDS-PAGE gel and western blot autoradiography film (antibody ICSM 35). Boxed lanes appear in Extended Data Fig. 1.

Silver-stained SDS-PAGE gel

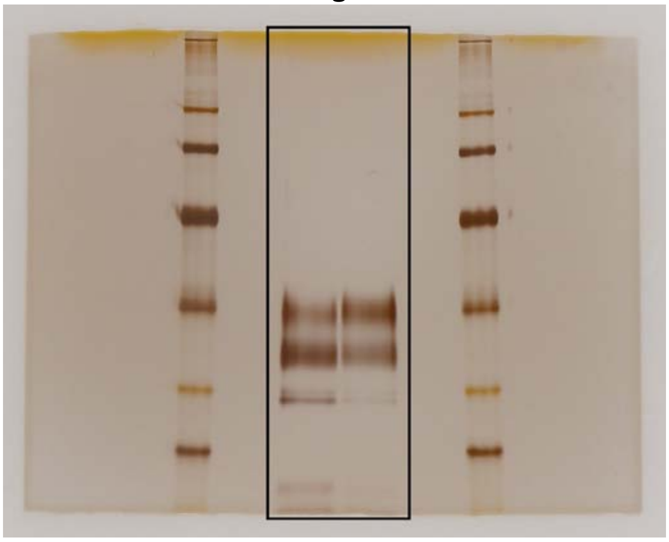

Western blot autoradiography film

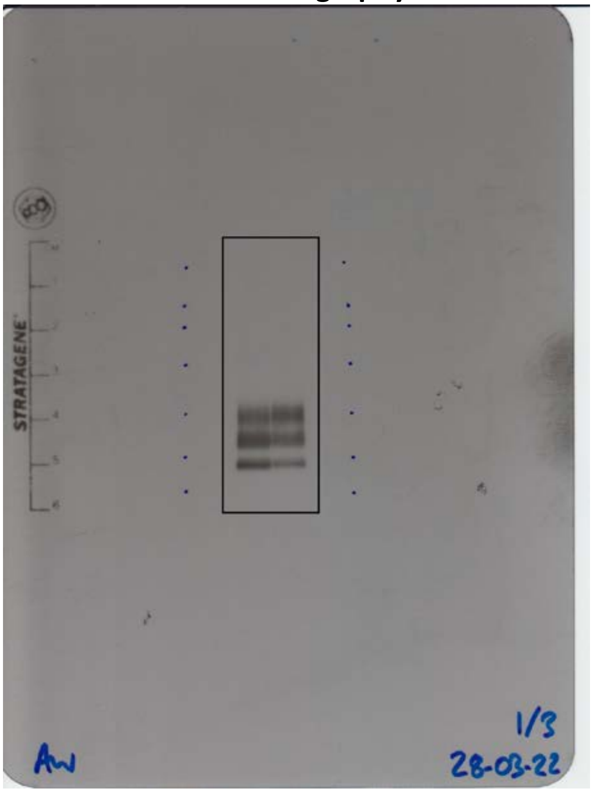

Supplement: Source Data Extended Data Fig. 1 — Unprocessed western blot and gel. [file 41589_2022_1229_MOESM3_ESM.pdf]
